# Unpacking the Social Media Happiness Paradox: How Social Media Stressors Undermine Subjective Well-Being Through Social Media Fatigue

**DOI:** 10.3390/bs16091551

**Published:** 2026-09-01

**Authors:** Yuzi Liu, Hua Pang, Yunze Zhao

**Affiliations:** 1 School of Journalism and Communication, Anhui University, Hefei 230601, China; liuyuziyz@outlook.com; 2 School of New Media and Communication, Tianjin University, Tianjin 300072, China; 3 School of Journalism, Renmin University of China, Beijing 100872, China

**Keywords:** social media happiness paradox, social media fatigue, subjective well-being, upward social comparison tendency, social media stressors

## Abstract

With the increasing embeddedness of social media in everyday life, digital platforms have become important spaces for interpersonal connection, self-presentation, and emotional experience. However, continuous social media engagement does not necessarily enhance users’ happiness; instead, it may coincide with psychological strain and lower subjective well-being (SWB), forming what this study refers to as the social media happiness paradox. In this study, the paradox is positioned as the focal phenomenon to be explained rather than as a standalone empirical construct. Drawing on social comparison theory and the stress-strain-outcome framework, this study examines the associations of social overload (SO), fear of missing out (FOMO), and upward social comparison tendency (USCT) with social media fatigue (SMF) and SWB, including statistical indirect associations through SMF. Based on 748 valid questionnaire responses, this study constructs an integrated conceptual model to examine the relationships among social media-related stressors, fatigue, and well-being. The findings show that SO, FOMO, and USCT are significantly and positively associated with SMF, while all three variables are significantly and negatively associated with SWB. In addition, significant statistical indirect associations between each stressor and SWB were observed through SMF. Taken together, the observed pattern is consistent with an integrated stressor–strain account of the social media happiness paradox and suggests directions for future digital well-being research and intervention design.

## 1. Introduction

In recent years, social media has become deeply embedded in almost every dimension of individual life, serving as a central platform for social connection, information acquisition, and self-presentation (Burrell & Fourcade, 2021). Social media platforms not only provide users with continuous opportunities for connectivity but are also embedded in the formation and maintenance of interpersonal relationships through large-scale information flows and interactive mechanisms (Reza et al., 2024). However, high-frequency social media use and sustained exposure to performative online content may also be associated with psychological strain (Firth et al., 2019). In the pursuit of social recognition and positive feedback, users are increasingly exposed to emotional exhaustion, psychological depletion, and social media fatigue (SMF) (Sheng et al., 2023). This tension highlights a central contradiction in the digital age: while social media is often used to enhance connection and happiness, particular patterns of use may simultaneously be associated with lower subjective well-being (SWB) (Silva, 2025). As social media platforms become increasingly widespread and functionally diversified, users are no longer merely passive consumers of content, but also active creators, interactants, and performers within digital social environments. Consequently, their psychological states and usage behaviors have become more complex and dynamic (Heinonen, 2011). This tension motivates the present study. In this article, the social media happiness paradox refers to the coexistence of connection- and gratification-oriented social media use with fatigue and lower subjective well-being (Gong, 2025; Mamgai & Kardam, 2023). The paradox serves as the focal phenomenon to be explained rather than as a standalone empirical construct.

Hedonic adaptation offers contextual background for why short-term positive feedback may not translate into sustained improvements in well-being (Diener et al., 2006; Sheldon et al., 2010). Because adaptation, reward seeking, and emotional monitoring are not directly measured, they are not treated as empirical mechanisms in the present model. The empirical analysis instead focuses on whether upward social comparison tendency (USCT), fear of missing out (FOMO), and social overload (SO) are associated with SMF and subjective well-being (SWB).

Despite the growing body of literature on the social media happiness paradox, social comparison theory, and social media fatigue, several important gaps remain. First, although existing studies have suggested that USCT, FOMO, and SO may be associated with higher SMF (Wang et al., 2023; Ou et al., 2023), these stressors have often been examined separately. It therefore remains important to determine whether they retain distinct associations with SMF when considered within the same conceptual model. Second, existing research has not sufficiently clarified whether SMF represents a common strain pathway linking these related but theoretically different stressors with SWB. Finally, although the association of USCT with psychological states and emotional experiences has received considerable scholarly attention (Jang et al., 2016; Park & Baek, 2018), fewer studies have positioned USCT alongside FOMO and SO as an evaluative stressor within the stressor-strain-outcome framework. In short, the research gap does not lie in the absence of evidence for the individual relationships. Rather, it lies in the need for an integrated account that distinguishes interactional overload, connectivity-related anxiety, and evaluative comparison and examines their direct and indirect associations with SWB. Specifically, the study addresses the following questions.

RQ1: How are social overload, fear of missing out, and upward social comparison tendency associated with social media fatigue when considered within the same conceptual model?

RQ2: How are social overload, fear of missing out, upward social comparison tendency, and social media fatigue associated with users’ subjective well-being?

RQ3: Does social media fatigue statistically mediate the associations between social overload, fear of missing out, upward social comparison tendency, and subjective well-being?

It is argued that the current investigation makes substantial contributions to the prevailing literature concerning the social media happiness paradox, SMF and SWB by examining users’ psychological resource depletion within digital social environments. First, by extending happiness paradox theory to the context of social media use, this study explores how users’ pursuit of connection, recognition, and emotional gratification may paradoxically be associated with fatigue and lower SWB. Recent research has shown that social media-related stressors, such as privacy concerns, time cost, overload, and emotional exhaustion, can significantly contribute to users’ social media fatigue and subsequent disengagement from social media interaction (Baj-Rogowska, 2023; Sheng et al., 2023). By elucidating the roles of USCT, FOMO and SO through the lens of psychological resource depletion, this study provides fresh theoretical insights into the patterns through which positive use motivations may coexist with negative psychological outcomes in digital social interaction. Second, this research constructs an integrated theoretical model to examine the direct and indirect pathways linking USCT, FOMO, SO, SMF, and SWB. Recent evidence suggests that social overload has been examined as a mediating pathway between FOMO and social media fatigue, indicating that users’ fear of being excluded from online social life may be associated with fatigue in the context of excessive social demands and interactional burden (Xie et al., 2024). This framework not only clarifies how multiple social media-related stressors are jointly associated with SMF but also provides evidence of a statistical indirect role of SMF in the relationship between these antecedent variables and users’ SWB. In doing so, the study provides a replicable analytical pathway for future research on digital well-being, social media fatigue, and the psychological consequences of platform-based interaction. Third, recognizing the limited prior exploration of how different stressors embedded in social media environments are collectively associated with users’ psychological outcomes, this study further analyzes the pattern of associations surrounding SMF and its negative association with SWB. This analysis offers practical guidance for healthier social media platform design and user behavior interventions by identifying comparison pressure, FOMO, and SO as potential targets for future intervention research. Ultimately, the findings underscore the relevance of the social media happiness paradox for understanding more sustainable patterns of social media use in the digital age.

## 2. Literature Review and Research Hypotheses

### 2.1. The Paradoxical Relationship Between Social Media Use and SWB in the Digital Age

The paradox of happiness refers to the possibility that intensified pursuit of, or investment in, happiness may paradoxically be associated with lower SWB (Irmer & Schmiedek, 2023; Zerwas et al., 2024). The Easterlin Paradox illustrates this tension at the societal level, showing that higher national income does not necessarily correspond to equivalent gains in average happiness (Demougin & Upton, 2023). Psychological research similarly suggests that treating happiness as an outcome that must be achieved may heighten frustration and emotional pressure (Humphrey et al., 2022). Social media provides a particularly salient context because users commonly seek connection, pleasure, self-expression, and recognition, while passive and comparison-oriented use is more consistently associated with lower SWB, and evidence concerning active use remains mixed (Uzcátegui-Sánchez et al., 2025; Valkenburg et al., 2022). In the present study, the social media happiness paradox is therefore used as an overarching conceptual lens rather than as a separately measured construct. The proposed model examines one specific stressor–strain pattern through which social media engagement may be associated with fatigue and lower SWB.

Hedonic adaptation provides contextual background for understanding why the short-term rewards of social media may not produce sustained improvements in well-being. Individuals tend to adapt to both positive and negative experiences (Höffken, 2025), and intensive monitoring of whether one feels sufficiently happy may generate disappointment or self-criticism (Huang, 2024; Brown & Rohrer, 2020). In social media settings, likes, comments, and follower growth may provide immediate gratification, but the adaptation process itself is not directly measured in this study. The operational account instead draws on conservation of resources theory, which suggests that stress arises when resources are lost, threatened, or insufficiently replenished after investment (Hollebeek et al., 2023), and on the stressor–strain–outcome framework (Koeske & Koeske, 1993). Accordingly, SO, FOMO, and USCT are conceptualized as three distinct stressors, SMF as psychological strain, and SWB as the focal outcome. Idealized self-presentation is retained as a contextual feature that may intensify external-validation demands and comparison, rather than as an additional empirical construct.

Maintaining an idealized online image may create emotional dissonance between online presentation and offline experience (Hsu et al., 2024). Exposure to selectively positive portrayals may also heighten perceived discrepancies between users’ own lives and those of others, increasing sensitivity to FOMO and upward comparison (Jabeen et al., 2023). At the same time, creating and maintaining idealized content requires emotional, cognitive, and temporal investment, while the perceived rewards may be short-lived. This resource imbalance offers a theoretical interpretation of why social media engagement may coexist with exhaustion, although the resource-loss process is not directly tested. Meta-analytic evidence nevertheless identifies social overload, anxiety, and compulsive use as important correlates of SMF (Ou et al., 2023). These considerations establish the theoretical background for the hypotheses developed in Section 2.2 and Section 2.3. The model’s incremental value lies in distinguishing interactional overload, connectivity-related anxiety, and evaluative comparison and examining whether they retain distinct associations with SMF when estimated simultaneously, as well as whether SMF forms a common statistical indirect pathway to SWB.

### 2.2. From Gratification-Seeking to Social Media Fatigue: A Uses and Gratifications Perspective

Uses and Gratifications Theory provides relevant theoretical background for interpreting social media fatigue. This framework regards users as active agents who selectively use media to satisfy specific needs and motivations, including entertainment, information seeking, social interaction, and emotional support (Bowden-Green et al., 2021). In the highly feedback-dependent environment of social media, however, such gratification may be temporary and may coexist with psychological costs, emotional emptiness, and fatigue (McLean et al., 2025). This perspective helps position self-presentation as one form of active and instrumental media use, although self-presentation itself is not tested in the empirical model (de Segovia Vicente et al., 2024; Pit et al., 2022). However, this effort to construct a positive self-image marks a potential source of contradiction. Investment in performative self-presentation and a perceived mismatch between input and reward may be associated with negative cognitive evaluations and SMF. These proposed processes are not directly measured in the present study.

On the basis of this motivational dissonance, the structural affordances of social media may be associated with two distinct forms of stress related to fatigue, namely FOMO and SO (Üztemur et al., 2025). FOMO primarily reflects connectivity-related anxiety arising from the perceived possibility of missing socially rewarding experiences, whereas SO reflects the interactional burden created by excessive social demands and relationship-maintenance obligations. FOMO refers to an anxious psychological state characterized by the fear that one may miss rewarding experiences in which others are participating (Tanhan et al., 2022). It is commonly linked to individuals’ basic need for belonging and social acceptance. On social media, FOMO may be associated with compulsive and persistent connectivity to avoid missing important information or interactions. This anxiety may be associated with a shift from active and instrumental social media use towards a passive, habitual, and even addictive pattern of use (Lopes et al., 2022). Such anxiety-associated use may involve a sustained investment of psychological resources, although this process is not directly observed in the present data. SO refers to a condition in which the number of social relationships users are expected to manage and maintain, together with the information flows generated by these relationships, exceeds their cognitive and temporal processing capacities (Zhang et al., 2022). As online connections expand and interactional demands increase, SO and information overload may be associated with greater social pressure and SMF, which is manifested in boredom, avoidance, reduced willingness to engage, and discontinuous usage intention (Ou et al., 2023). Thus, the following hypotheses are proposed:

**H1.**  *Social overload is positively associated with social media fatigue.*

**H2.**  *Fear of missing out is positively associated with social media fatigue.*

### 2.3. Upward Social Comparison, Social Media Fatigue, and Subjective Well-Being

Social comparison is a universal psychological tendency through which individuals evaluate their abilities, opinions, and social standing by comparing themselves with others (Derbaix et al., 2025; Irmer & Schmiedek, 2023). In social media environments, however, this tendency may be associated with platform mechanisms such as visibility, curation, and algorithmic exposure (Nor et al., 2025). Social comparison theory therefore provides a direct psychological framework for interpreting why users may experience negative self-evaluations and emotional strain when they are repeatedly exposed to performative online content (McComb et al., 2023; Zhao & Bai, 2025). Social media provides users with frequent and selectively curated comparison targets, which may increase opportunities for USCT. Reliance on external comparison standards may also be associated with less stable self-evaluation and well-being (Bırni & Eryılmaz, 2024).

Under such conditions, users may increasingly evaluate themselves according to whether they appear more successful than the comparison targets presented online. Their attention may consequently shift from intrinsic goals toward avoiding perceived inferiority or failure in comparison. The pressure and anxiety that users experience within this comparison-based environment are consistent with a psychological interpretation of the social media happiness paradox. When comparison pressure coincides with envy or anxiety, it may also be associated with disengagement behaviors such as reduced use or disabled notifications (Cao et al., 2025). Although upward comparison may sometimes motivate self-improvement, exposure to selectively positive targets may also be associated with contrastive self-evaluations and negative emotions (Arigo et al., 2024).

Users who compare their complex lives with idealized portrayals of others may report frustration and relative deprivation. USCT is not merely a cognitive comparison process; it may also be accompanied by negative affective responses, such as envy, anxiety, and diminished self-evaluation. These responses may be associated with greater psychological strain and less favorable evaluations of well-being (Irmer & Schmiedek, 2023). Social comparison theory thus offers a relevant psychological interpretation of the social media happiness paradox. Repeated upward comparison may coexist with anxiety, emotional demands, and greater vulnerability to SMF. Within the integrated model, USCT represents an evaluative stressor that differs from the interactional burden captured by SO and the connectivity-related anxiety captured by FOMO. Examining these three stressors simultaneously makes it possible to assess whether comparison pressure retains a distinct association with SMF and whether the model supports statistical indirect associations through SMF. Therefore, this study proposes the following hypotheses:

**H3.**  *Upward social comparison tendency is positively associated with social media fatigue.*

**H4.**  *The respective indirect associations of social overload, fear of missing out, and upward social comparison tendency with subjective well-being through social media fatigue are statistically significant.*

**H5.**  *Upward social comparison tendency, social overload, fear of missing out, and social media fatigue are negatively associated with subjective well-being.*

**H5a.**  *Upward social comparison tendency is negatively associated with subjective well-being.*

**H5b.**  *Social overload is negatively associated with subjective well-being.*

**H5c.**  *Fear of missing out is negatively associated with subjective well-being.*

**H5d.**  *Social media fatigue is negatively associated with subjective well-being.*

## 3. Research Design

### 3.1. Research Model

**Figure 1:**
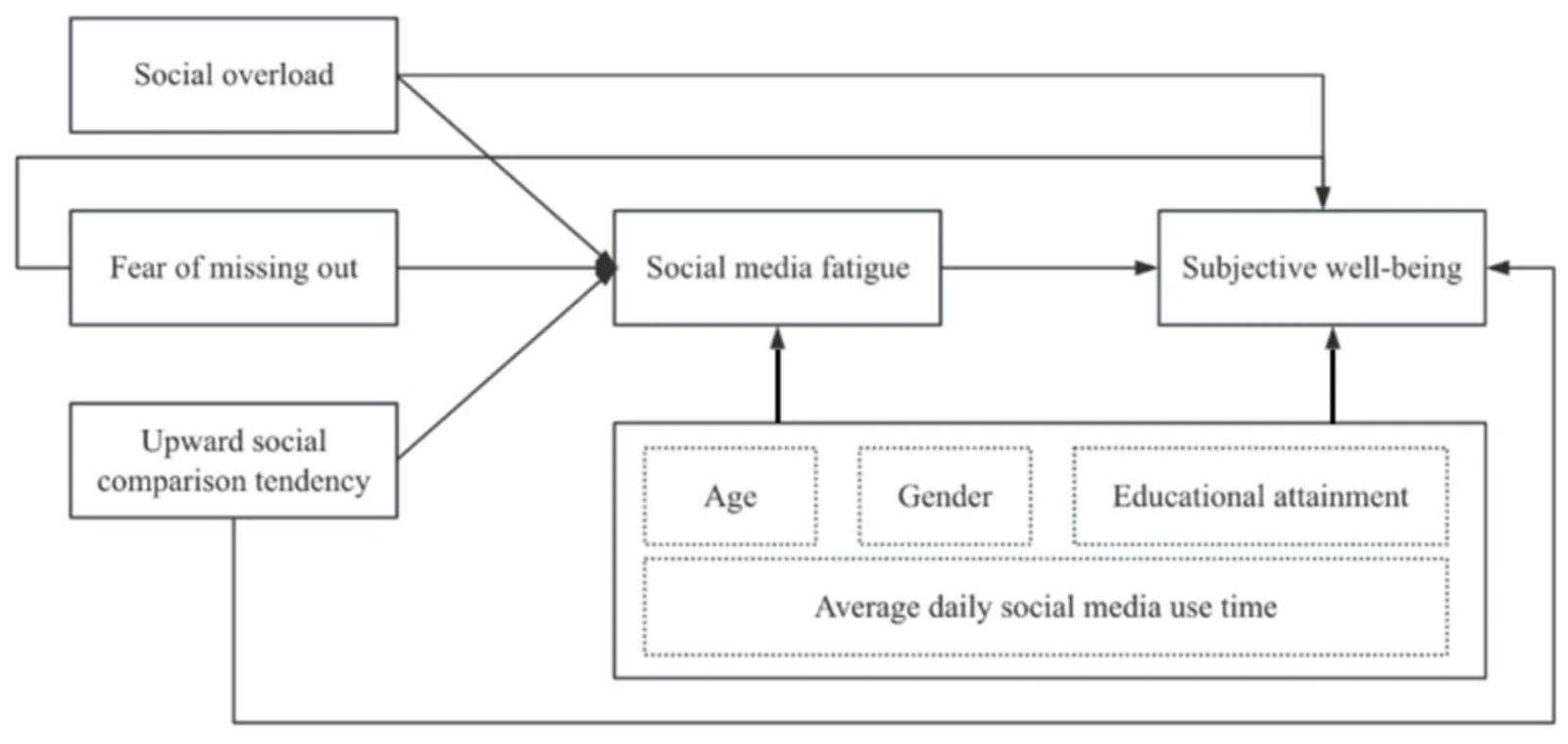
The conceptual research model.

This study focuses on several core variables, including USCT, SO, FOMO, SMF, and SWB. It examines the direct and indirect associations of these factors with individuals’ SWB. Specifically, USCT, SO, and FOMO are modeled as distinct social media-related stressors, while SMF is modeled as a psychological strain that may be statistically involved in their relationships with SWB. On this basis, the study constructs a research framework, as shown in Figure 1, to examine one stressor–strain pattern related to the social media happiness paradox and users’ SWB. This framework not only clarifies the relationships among the key variables but also provides a theoretical basis for understanding why social media use may simultaneously bring social connection and psychological burden to users. To improve the robustness of the proposed model, age, gender, educational attainment, and average daily social media use time were included as control variables. These variables were specified as predictors of social media fatigue and subjective well-being in the structural model. Age, gender, and educational attainment were included to account for potential demographic differences in social media engagement and well-being, while average daily social media use time was included to account for the general intensity of platform exposure. This approach helps reduce the possibility that the hypothesized relationships are confounded by basic demographic differences or general intensity of social media use. Given the cross-sectional design, the directional paths in Figure 1 represent theory-guided associations rather than temporal or causal effects.

### 3.2. Data Collection

The questionnaire consisted of three sections. The first section collected demographic information, including gender, age, educational attainment, occupational status, years of work experience, and current city of residence. The work-experience item was displayed only to respondents who identified themselves as employed professionals; therefore, 314 respondents completed this item, while the item was not applicable to the remaining respondents. The second section measured media-use behaviors, including social media use tendency, average daily usage time, typical usage behaviors, and frequency of switching across platforms. The third section measured the main constructs of this study, including USCT, FOMO, SO, SMF, and SWB.

Among these variables, age, gender, educational attainment, and average daily social media use time were used as control variables in the subsequent analyses.

### 3.3. Measurement

The three USCT items were adapted with reference to the social-media upward-comparison literature summarized in (McComb et al., 2023). The three-item FOMO measure drew on the theoretical and empirical work of (Przybylski et al., 2013), while the three-item SMF measure was adapted from (Qin et al., 2024). Each of the five focal constructs was measured with three items, for a total of 15 construct items. The measurement items for all constructs were mainly adapted from mature scales in existing research and were revised in accordance with the usage characteristics of contemporary social media platforms to ensure reliability and validity. To reduce response bias caused by respondents’ potential misunderstanding of item meanings, complex expressions in the questionnaire were replaced with more accessible wording while maintaining semantic consistency. Because the original scales were developed in English, the items were translated into Chinese and independently back translated into English. Differences between the original and back-translated versions were reviewed and resolved through discussion to ensure linguistic and conceptual equivalence. Minor wording adjustments were then made to improve comprehensibility in the context of contemporary Chinese social media use without changing the substantive meaning of the items. In addition, a self-assessment item was included at the end of the questionnaire: “I felt that this questionnaire was quick to complete and did not involve any difficulty in understanding.” This item was used to evaluate the overall comprehensibility and ease of completion of the questionnaire. All construct variables were measured using a five-point Likert scale, with 1 indicating “strongly disagree” and 5 indicating “strongly agree.” Before the main recruitment phase, more than 50 early responses were reviewed to assess questionnaire comprehensibility, item wording, and completion burden. Only minor wording refinements were made, without changing the substantive meaning, number, response format, or structure of the measurement items. Because these early responses were collected using substantively the same questionnaire and were retained in the final analytical sample, this stage is described as an initial questionnaire check rather than as an independent pilot study. The formal survey was conducted online. Data collection was carried out through ‘Wenjuanxing (Sojump)’, a widely used online questionnaire platform in China, between 25 October and early December 2025. Participants were recruited primarily through WeChat groups and social media sharing. Eligible participants were required to be at least 18 years old, to use social media regularly, and to provide voluntary informed consent before completing the questionnaire. No monetary or material incentives were provided. A total of 800 completed questionnaires were collected. Because recruitment relied on voluntary online participation and social-media-based dissemination, the sampling procedure was non-probabilistic rather than random.

### 3.4. Data Screening and Statistical Analysis

During data preprocessing, questionnaire quality was assessed using completion-time checks and manual review of response patterns and logical consistency. A response was excluded when it met one or more of the following criteria: a completion time of less than 60 s, selection of the same response option for more than 90% of the substantive questionnaire items, or logically inconsistent answers across related questions. Because these screening criteria could overlap, each excluded respondent was counted only once, and no mutually exclusive breakdown by exclusion reason was calculated. A total of 52 responses were removed, leaving 748 valid questionnaires and a valid-response rate of 93.5%. The detailed demographic characteristics of the sample are presented in Table 1. The work-experience item was conditionally displayed only to respondents who identified themselves as employed professionals. Consequently, this variable included 314 valid responses, and its percentages were calculated using this subgroup rather than the full sample. The remaining 434 respondents were not presented with the item and were coded as not applicable rather than missing.

After data screening, descriptive analyses were conducted to summarize respondents’ demographic characteristics and social media use patterns. Pearson product–moment correlation analyses were then performed to examine the zero-order associations among the control variables and key constructs. Potential multicollinearity among the predictors was assessed using variance inflation factors.

Subsequently, the hypothesized five-factor measurement model was evaluated using confirmatory factor analysis within a covariance-based structural equation modeling framework. Overall model fit was assessed using the chi-square statistic, the chi-square-to-degrees-of-freedom ratio, RMSEA with its 90% confidence interval, CFI, TLI, and SRMR. Standardized factor loadings, Cronbach’s α, composite reliability, and average variance extracted were examined to assess construct reliability and convergent validity. Discriminant validity was evaluated using the Fornell–Larcker criterion and the heterotrait–monotrait ratio of correlations.

Potential common method variance was assessed by comparing the hypothesized five-factor model with a single-factor model and by estimating an additional common latent method factor. In the common latent method factor model, all items loaded on both their respective substantive constructs and an orthogonal method factor. The unstandardized method-factor loadings were constrained to equality for model identification, and the scale of the method factor was established by fixing its variance to 1. Interpretation focused on whether adding the method factor produced a substantive improvement in model fit; only small changes in fit indices were taken as evidence that a general method factor was unlikely to dominate the covariance structure. Conceptually, the method factor represents residual covariance shared across all 15 items after the five substantive constructs are taken into account; it is a diagnostic for a general method-related component rather than a measure of any specific response bias. Although standardized coefficients can be mechanically generated, the present identification strategy estimates only one equality-constrained unstandardized method-loading parameter. The resulting standardized coefficients would therefore be rescaled versions of that single parameter as a function of each item’s total variance, rather than independently estimated item-specific method effects. Reporting them as separate substantive loadings could imply item-level information that the model does not estimate, so they were not interpreted or reported separately. The fixed method-factor variance of 1 serves only to establish the factor’s scale and cannot be interpreted as the percentage of observed variance attributable to method effects. Accordingly, the CMV assessment relied on converging model-level evidence from the single-factor comparison and the incremental fit of the common-method-factor model. The conclusion was deliberately limited to whether a general method factor dominated the covariance structure; the analysis was not treated as evidence that all possible forms of CMV were absent. Subsequently, the hypothesized direct and indirect relationships were estimated simultaneously within a latent-variable structural equation model. Age, gender, educational attainment, and average daily social media use time were included as predictors of social media fatigue and subjective well-being. Indirect effects were evaluated using bias-corrected bootstrap confidence intervals based on 5000 resamples.

Data screening, descriptive statistics, reliability analysis, correlation analysis, and multicollinearity diagnostics were conducted using SPSS 26.0. Confirmatory factor analysis and structural equation modeling were performed using AMOS 26.

## 4. Results

### 4.1. Measurement Model Assessment

The five-factor measurement model was assessed using confirmatory factor analysis within a covariance-based structural equation modeling framework. The model showed an acceptable fit to the data: χ^2^ = 217.521, *df* = 80, χ^2^/*df* = 2.719, RMSEA = 0.048, 90% CI [0.040, 0.056], CFI = 0.928, TLI = 0.911, and SRMR = 0.061. Taken together, these indices indicated that the proposed five-factor structure provided an adequate representation of the observed covariance matrix.

As shown in Table 2, the standardized factor loadings ranged from 0.635 to 0.863 and were all statistically significant at *p* < 0.001. The construct-specific Cronbach’s α coefficients ranged from 0.793 to 0.889, while the composite reliability values ranged from 0.801 to 0.890. The average variance extracted values ranged from 0.504 to 0.652. These results supported the internal consistency and convergent validity of the five construct measures.

Discriminant validity was first assessed using the Fornell–Larcker criterion. As shown in Table 3, the square root of each construct’s AVE exceeded the absolute value of its correlations with the other constructs. As an additional assessment, the HTMT values ranged from 0.612 to 0.842, with the highest value observed between upward social comparison tendency and subjective well-being. Although this value approached the conservative threshold of 0.85, all HTMT values remained below it. Taken together, the Fornell–Larcker and HTMT results provided acceptable support for the discriminant validity of the five constructs.

### 4.2. Correlation and Multicollinearity Analysis

Pearson product–moment correlation analysis was conducted to examine the zero-order associations among the control variables and key constructs. As shown in Table 4, the correlations among the main constructs were consistent with theoretical expectations. Social overload was positively correlated with fear of missing out (*r* = 0.454, *p* < 0.01), upward social comparison tendency (*r* = 0.596, *p* < 0.01), and social media fatigue (*r* = 0.436, *p* < 0.01), while it was negatively correlated with subjective well-being (*r* = −0.602, *p* < 0.01). Fear of missing out was positively associated with upward social comparison tendency (*r* = 0.588, *p* < 0.01) and social media fatigue (*r* = 0.503, *p* < 0.01) but negatively associated with subjective well-being (*r* = −0.605, *p* < 0.01). Upward social comparison tendency was positively associated with social media fatigue (*r* = 0.556, *p* < 0.01) and negatively associated with subjective well-being (*r* = −0.705, *p* < 0.01). Social media fatigue was also negatively associated with subjective well-being (*r* = −0.588, *p* < 0.01). Overall, the correlation pattern was directionally consistent with the proposed relationships among social media-related stressors, fatigue, and subjective well-being.

To assess whether the comparatively high correlations among several constructs resulted in problematic multicollinearity, variance inflation factors were calculated for the predictor sets associated with the two endogenous variables. When social media fatigue was specified as the outcome, the VIF values for social overload, fear of missing out, and upward social comparison tendency were 1.61, 1.60, and 1.93, respectively. When subjective well-being was specified as the outcome, the VIF values for social overload, fear of missing out, upward social comparison tendency, and social media fatigue were 1.62, 1.69, 2.11, and 1.60, respectively. The VIF values for the control variables ranged from 1.01 to 1.09. These results indicated that multicollinearity was unlikely to materially affect the structural path estimates.

### 4.3. Common Method Variance Assessment

The single-factor model showed a substantially poorer fit than the hypothesized five-factor model: χ^2^ (90) = 1120.38, *p* < 0.001, χ^2^/*df* = 12.45, RMSEA = 0.124, 90% CI [0.117, 0.130], CFI = 0.481, TLI = 0.395, and SRMR = 0.139. This result indicated that a single common factor could not adequately account for the covariance among the measurement items.

The common latent method factor model showed only a marginal improvement in fit: χ^2^ (79) = 211.20, *p* < 0.001, χ^2^/*df* = 2.67, RMSEA = 0.047, 90% CI [0.040, 0.055], CFI = 0.931, TLI = 0.912, and SRMR = 0.059. Relative to the five-factor model, these changes were small (ΔCFI = 0.003, ΔTLI = 0.001, ΔRMSEA = −0.001, and ΔSRMR = −0.002). The nested comparison yielded Δχ^2^ (1) = 6.32, *p* = 0.012. Although this chi-square difference was statistically significant, the approximate fit indices improved by only 0.001–0.003, indicating little practical improvement in the model’s representation of the covariance structure. Standardized item-level method-factor loadings were not reported because the model estimated only one equality-constrained unstandardized method-loading parameter; any standardized coefficients would be rescaled expressions of that single parameter and item variances, not independently estimated item-specific effects. The CMV judgment was therefore based on model-level evidence: the single-factor model fit the data very poorly, whereas adding the general method factor to the five-factor model produced negligible changes in CFI, TLI, RMSEA, and SRMR. Taken together, this pattern indicates that a general common-method factor did not materially improve the measurement model and was unlikely to dominate the covariance structure. This conclusion does not establish the absence of CMV, which remains a limitation of the cross-sectional self-report design.

### 4.4. Structural Model and Hypothesis Testing

The hypothesized relationships were estimated simultaneously using covariance-based structural equation modeling. Social overload, fear of missing out, and upward social comparison tendency were specified as predictors of both social media fatigue and subjective well-being, while social media fatigue was included as an additional predictor of subjective well-being. Age, gender, educational attainment, and average daily social media use time were included as control variables predicting the two endogenous constructs.

The structural model showed an acceptable fit to the data: χ^2^ (128) = 338.41, *p* < 0.001, χ^2^/*df* = 2.64, RMSEA = 0.047, 90% CI [0.041, 0.053], CFI = 0.923, TLI = 0.908, and SRMR = 0.064. The model explained 43.7% of the variance in social media fatigue and 68.1% of the variance in subjective well-being.

As shown in Table 5, social overload was positively associated with social media fatigue (β = 0.183, *p* < 0.001), supporting H1. Fear of missing out was also positively associated with social media fatigue (β = 0.276, *p* < 0.001), supporting H2. Upward social comparison tendency showed a significant positive association with social media fatigue (β = 0.331, *p* < 0.001), supporting H3.

Regarding subjective well-being, upward social comparison tendency showed a significant negative association with subjective well-being (β = −0.354, *p* < 0.001), supporting H5a. Social overload (β = −0.196, *p* < 0.001) and fear of missing out (β = −0.172, *p* < 0.001) were also negatively associated with subjective well-being, supporting H5b and H5c, respectively. In addition, social media fatigue was negatively associated with subjective well-being (β = −0.238, *p* < 0.001), supporting H5d. Taken together, the results supported all hypothesized direct associations.

The indirect effects were subsequently assessed using bias-corrected bootstrapping with 5000 resamples. As shown in Table 6, the indirect association between social overload and subjective well-being through social media fatigue was significant (β = −0.044, 95% CI [−0.071, −0.018]). The indirect association between fear of missing out and subjective well-being through social media fatigue was also significant (β = −0.066, 95% CI [−0.099, −0.038]). Similarly, upward social comparison tendency had a significant indirect association with subjective well-being through social media fatigue (β = −0.079, 95% CI [−0.117, −0.047]). None of the bootstrap confidence intervals included zero.

**Figure 2:**
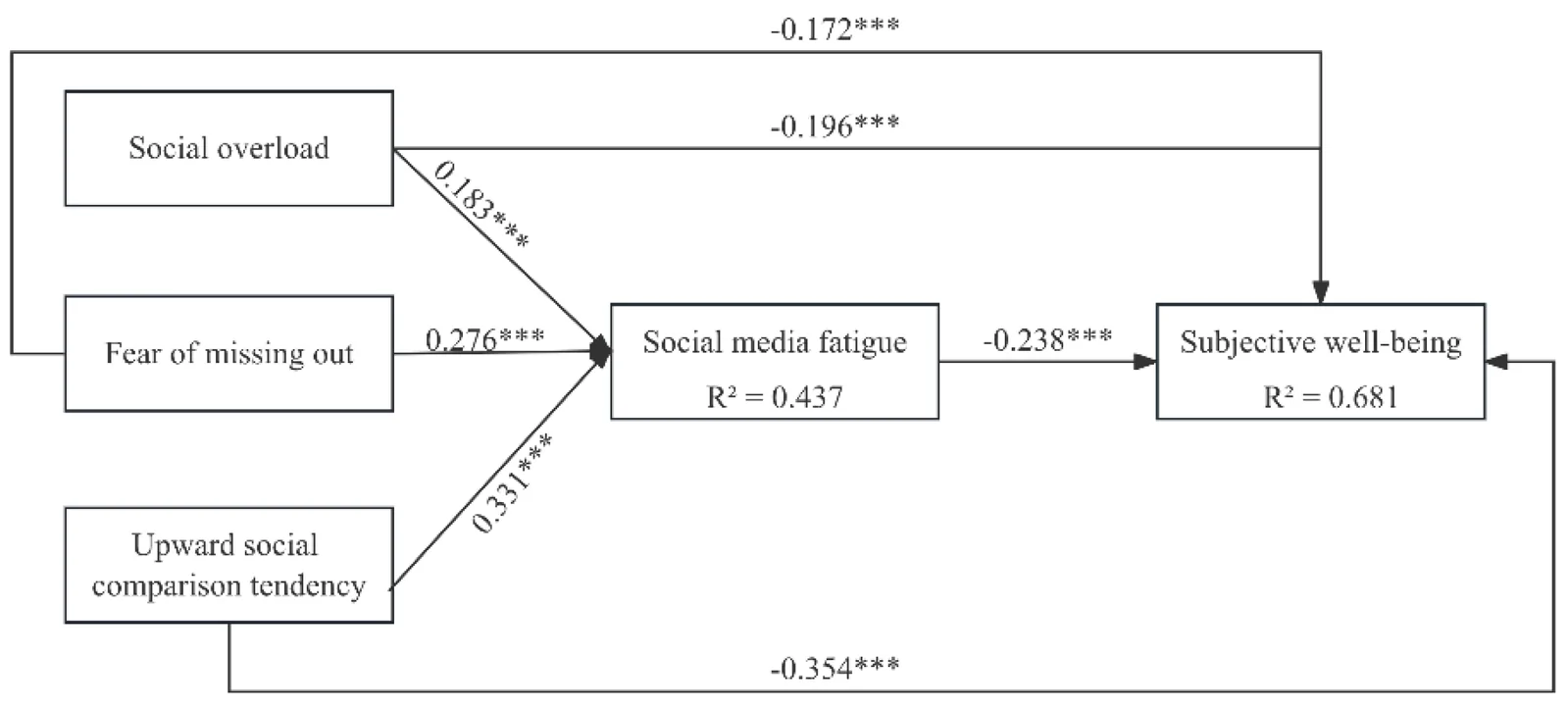
Standardized path coefficients are reported. Age, gender, educational attainment, and average daily social media use time were included as control variables but are omitted from the figure for visual clarity. *** *p* < 0.001.

Because the direct associations of social overload, fear of missing out, and upward social comparison tendency with subjective well-being remained statistically significant after social media fatigue was included in the model, social media fatigue statistically mediated, but did not fully account for, these associations. Therefore, H4 was supported. The standardized structural coefficients are presented in Figure 2.

## 5. Discussion

### 5.1. Conclusions

The results identify a coherent stressor–strain pattern associated with the social media happiness paradox. SO, FOMO, and USCT were each positively associated with SMF and negatively associated with SWB, while SMF was negatively associated with SWB (Zhu & Yao, 2025). All three stressors also showed significant indirect associations with SWB through SMF. These relationships remained evident after age, gender, educational attainment, and average daily social media use time were included as controls. Thus, the findings indicate that interactional overload, connectivity-related anxiety, and evaluative comparison are distinguishable correlates of fatigue and lower well-being within the same model.

SO and FOMO retained distinct positive associations with SMF when modeled simultaneously, supporting their conceptual differentiation rather than their treatment as interchangeable indicators of general social media stress. USCT also retained a distinct association with SMF, extending prior work that has primarily connected upward comparison with self-evaluation and well-being (McComb et al., 2023). Because resource depletion, self-efficacy loss, and other intervening processes were not directly measured, they should remain theoretical interpretations rather than demonstrated mechanisms.

SMF statistically mediated, but did not fully account for, the associations of SO, FOMO, and USCT with SWB. This pattern is consistent with the stressor–strain–outcome framework: conceptually different social media-related demands may converge on fatigue as a shared strain while retaining separate associations with well-being. The results do not demonstrate interactions among the three stressors, and the indirect effects do not establish causal mediation. Because all variables were measured at one point in time, longitudinal or experimental evidence is required to determine temporal ordering and causality.

### 5.2. Theoretical and Practical Implications

The findings offer three related theoretical implications. First, they specify one empirical pattern consistent with the social media happiness paradox without treating the paradox itself as an independently measured construct. Second, the simultaneous estimation of SO, FOMO, and USCT distinguishes interactional overload, connectivity-related anxiety, and evaluative comparison while showing that each retains a separate association with SMF. Third, positioning USCT as an evaluative stressor connects social comparison theory with the stressor–strain–outcome framework, while the indirect associations identify SMF as a common statistical strain pathway. The contribution therefore lies in the integration and differentiation of these stressors within one model, rather than in presenting each individual association as entirely novel. The findings also identify FOMO, SO, USCT, and SMF as potential targets for future digital well-being interventions. The platform and policy proposals discussed below should be understood as applications derived from the observed associations rather than as interventions directly tested by the present cross-sectional study.

First, given the observed associations of FOMO, social overload, and upward comparison with fatigue, intervention strategies may prioritize the cultivation of psychological resilience and the reconstruction of cognitive boundaries. Educational institutions and psychological counseling organizations should develop emotional resilience training programs tailored to digital media environments. Through cognitive behavioral approaches, such programs could be designed to help users identify and reconsider comparison-related envy, shifting the cognitive focus of comparison from self-devaluation to constructive self-improvement. At the same time, users should be guided to recognize their media-use motivations and emotional responses, with the aim of reducing anxiety-related patterns of compulsive use. Considering the high psychological cost of passive consumption, users should also be encouraged to engage in purposeful media use. Setting clear usage goals and time limits may encourage more purposeful media use; whether such strategies reduce negative outcomes associated with passive browsing should be tested directly (Mujica et al., 2022). Second, at the level of platform design and operation, social media platforms, as key architects of the media ecosystem, should assume greater social responsibility for reducing users’ psychological burden. Platform designers may monitor patterns of prolonged passive consumption and provide timely interventions when users remain immersed in performative content for extended periods. Such interventions could prompt users to reflect on their current motivations for use. To address compulsive use that may accompany FOMO, platforms could introduce functions such as batch notification processing and scheduled silent periods, encouraging users to shift their attention from immediate external prompts to autonomous choice and planning. To address SO, platforms could further improve tools for managing social relationships and interactional obligations, including options for limiting low-priority notifications or organizing contacts according to interaction preferences. These design suggestions should be understood as testable intervention proposals rather than as effects established by the present cross-sectional survey. Future experimental research is needed to determine whether such features reduce fatigue or improve SWB. Finally, public education may help users understand the selective and performative nature of social media content. Digital literacy education could be expanded to include awareness of idealized self-presentation, upward comparison, attention management, and emotional responses to platform use. Such initiatives may help users interpret online content more critically and establish more sustainable media-use practices. Broader institutional or policy interventions should likewise be evaluated before implementation, particularly because their effects may vary across user groups, platforms, and cultural contexts. Through coordinated efforts at the individual, platform, and educational levels, it may be possible to reduce unnecessary social media-related demands and support healthier patterns of digital engagement.

## 6. Limitations and Future Research

Although this study constructs a relatively comprehensive model for explaining the social media happiness paradox, several limitations remain in terms of theoretical mechanism, data collection, and content differentiation. First, regarding the proposed relationships, this study mainly examines the direct and indirect associations of USCT, FOMO, and SO with SMF and SWB. However, the internal psychological processes underlying each pathway, as well as potential moderating factors, have not been fully examined. In addition, the cross-sectional design does not permit firm conclusions regarding temporal order or causality, and reverse or reciprocal relationships cannot be ruled out. Future research could adopt longitudinal designs or experimental methods to clarify the temporal ordering and potential causal relationships among these variables and to capture more accurately the dynamic evolution of the happiness paradox in social media use. Second, in terms of data collection, this study adopted an online questionnaire method and included only Chinese users to maintain methodological consistency. Because participants were recruited through voluntary online participation and social-media-based dissemination, the resulting non-probability sample may overrepresent individuals who are more active on social media or more willing to participate in online surveys. This sampling strategy may limit the generalizability of the findings to some extent since users’ interaction behaviors and experiences of well-being may be shaped by cultural background, social norms, and platform-use environments. Moreover, all principal variables were measured using self-reported data collected from the same respondents at one point in time. Although the common method variance assessment did not suggest that a general method factor dominated the covariance structure, common method bias cannot be completely excluded. Self-reported estimates of social media use may also differ from actual usage behavior. Future studies could conduct empirical investigations in different countries and regions to examine whether the proposed model is applicable across diverse cultural contexts. They could also combine questionnaire data with objective indicators, such as device-recorded screen time, platform-use logs, notification frequency, or behavioral trace data. Third, regarding content dimensions, this study mainly focused on idealized self-presentation content in a general sense and did not further distinguish among specific content types. Future research could refine content categories by differentiating material display, achievement presentation, emotional sharing, and lifestyle performance. Such distinctions would make it possible to identify more precisely the heterogeneous associations of different content types with psychological pressure and fatigue. Future studies could also examine whether these relationships differ across image-based, video-based, and text-based social media platforms. This would provide a stronger theoretical and empirical basis for understanding the long-term evolution of the happiness paradox in social media use.

## Figures and Tables

**Table 1:** Demographics of the research sample and social media usage (N = 748).

Variable	Category	Frequency (N)	Percentage (%)
Age	18–24 years	227	30.35
	25–30 years	405	54.14
	31–35 years	116	15.51
Gender	Male	236	31.55
	Female	512	68.45
Occupational status	Student	296	39.57
	Employed professional	314	41.98
	Freelancer	96	12.83
	Other	42	5.61
Educational attainment	Junior college	71	9.46
	Bachelor’s degree	278	37.16
	Master’s degree	240	32.09
	Doctoral degree	159	21.28
Years of work experience	Less than 3 years	121	38.54
	3–5 years	114	36.31
	6–10 years	73	23.25
	More than 10 years	6	1.91
Current city of residence	First-tier city	220	29.41
	Second-tier city	263	35.16
	Third-tier city	187	25
	Fourth-tier city or below	78	10.43
Average daily social media use	Less than 1 h	0	0.00
	1–2 h	27	3.61
	2–4 h	265	35.43
	More than 4 h	456	60.96
Social media use tendency	Primarily active posting and interaction	232	31.02
	Primarily passive browsing of others’ content	516	68.98

Notes: Percentages were calculated based on the full sample of 748 respondents, except for years of work experience. This item was displayed only to employed professionals, resulting in 314 valid responses.

**Table 2:** Measurement model results.

Construct	Item	CR	Cronbach’s α	AVE	Standardized Factor Loading
Social overload	SO1	0.890	0.850	0.576	0.758
	SO2				0.724
	SO3				0.804
Fear of missing out	FOMO1	0.882	0.799	0.652	0.846
	FOMO2				0.762
	FOMO3				0.822
Upward social comparison tendency	USCT1	0.801	0.881	0.504	0.635
	USCT2				0.863
	USCT3				0.732
Social media fatigue	SMF1	0.847	0.889	0.583	0.648
	SMF2				0.851
	SMF3				0.815
Subjective well-being	SWB1	0.802	0.793	0.579	0.849
	SWB2				0.742
	SWB3				0.678

Notes: Standardized loadings were obtained from the confirmatory factor analysis. All factor loadings were statistically significant at *p* < 0.001. CR = composite reliability; AVE = average variance extracted.

**Table 3:** Discriminant validity assessment based on the Fornell–Larcker criterion.

	SO	FOMO	USCT	SMF	SWB
SO	(0.759)				
FOMO	0.454 **	(0.807)			
USCT	0.596 **	0.588 **	(0.710)		
SMF	0.436 **	0.503 **	0.556 **	(0.763)	
SWB	−0.602 **	−0.605 **	−0.705 **	−0.588 **	(0.761)

Notes: Double asterisks (**) represents *p* < 0.01; SO, social overload; FOMO, fear of missing out; USCT, upward social comparison tendency; social media fatigue; SMF, social media fatigue; SWB, subjective well-being. Values in parentheses are the square roots of the AVE values.

**Table 4:** Zero-order correlations among control variables and key constructs.

Variables	1	2	3	4	5	6	7	8	9
1. Age	1								
2. Gender	0.031	1							
3. Educational attainment	0.084 *	0.026	1						
4. Average daily social media use time	−0.112 **	0.043	0.037	1					
5. Social overload	−0.058	0.052	−0.041	0.184 **	1				
6. Fear of missing out	−0.096 **	0.068	−0.066	0.207 **	0.454 **	1			
7. Upward social comparison tendency	−0.082 *	0.045	−0.052	0.163 **	0.596 **	0.588 **	1		
8. Social media fatigue	−0.087 *	0.061	−0.047	0.224 **	0.436 **	0.503 **	0.556 **	1	
9. Subjective well-being	0.105 **	−0.049	0.073 *	−0.176 **	−0.602 **	−0.605 **	−0.705 **	−0.588 **	1

Notes: An asterisk (*) represents *p* < 0.05; Double asterisks (**) represents *p* < 0.01.

**Table 5:** Structural model results.

Hypothesis	Structural Path	Standardized β	S.E.	C.R.	*p*	Result
H1	Social overload → Social media fatigue	0.183	0.036	5.083	<0.001	Supported
H2	Fear of missing out → Social media fatigue	0.276	0.038	7.263	<0.001	Supported
H3	Upward social comparison tendency → Social media fatigue	0.331	0.041	8.073	<0.001	Supported
H5a	Upward social comparison tendency → Subjective well-being	−0.354	0.039	−9.077	<0.001	Supported
H5b	Social overload → Subjective well-being	−0.196	0.035	−5.600	<0.001	Supported
H5c	Fear of missing out → Subjective well-being	−0.172	0.036	−4.778	<0.001	Supported
H5d	Social media fatigue → Subjective well-being	−0.238	0.040	−5.950	<0.001	Supported

Note: Standardized coefficients are reported. Age, gender, educational attainment, and average daily social media use time were included as predictors of social media fatigue and subjective well-being. S.E. = standard error; C.R. = critical ratio.

**Table 6:** Bootstrap estimates of indirect effects.

Indirect Pathway	Standardized Indirect Effect	95% Bias-Corrected CI
SO → SMF → SWB	−0.044	[−0.071, −0.018]
FOMO → SMF → SWB	−0.066	[−0.099, −0.038]
USCT → SMF → SWB	−0.079	[−0.117, −0.047]

Note: Bootstrap confidence intervals were based on 5000 resamples. An indirect effect was considered statistically significant when its 95% confidence interval did not include zero. SO = social overload; FOMO = fear of missing out; USCT = upward social comparison tendency; SMF = social media fatigue; SWB = subjective well-being.

## Data Availability

The original contributions presented in this study are included in the article. Further inquiries can be directed to the corresponding authors.
